# Arthroscopic anterior cruciate ligament reconstruction with and without tourniquet use: an updated systematic review and meta-analysis on clinical outcomes

**DOI:** 10.1186/s12891-024-08101-w

**Published:** 2024-12-05

**Authors:** Mahdieh Samei, Mahla Daliri, Masoumeh Sadeghi, Reza Ganji, Ali Parsa, Mohammad H. Ebrahimzadeh

**Affiliations:** 1 Orthopedic Research Center, Ghaem Hospital, Mashhad University of Medical Sciences, Mashhad, 91766-99199 Iran; 2 Department of Epidemiology, School of Health, Mashhad University of Medical Sciences, Mashhad, Iran; 3 American Hip Institute, Chicago, Illinois USA; 4 Department of Orthopedic Surgery, School of Medicine, North Khorasan University of Medical Sciences, Bojnurd, Iran

**Keywords:** Anterior cruciate ligament, Knee, Surgery, Tourniquet, Arthroscopy, Drain output

## Abstract

**Background:**

The use of a tourniquet is common during anterior cruciate ligament (ACL) reconstruction, offering convenience for the surgical procedure. However, the potential adverse effects of tourniquet use have gained increasing attention from clinical researchers. We conducted this systematic review and meta-analysis to compare the clinical outcomes of tourniquet application versus non-tourniquet approach during arthroscopic ACL reconstruction.

**Methods:**

A comprehensive search of PubMed, Web of Science, Embase, and Cochrane Library databases, was performed through March 2023 to identify controlled clinical trials. The main outcomes assessed included post-operative drain output, post-operative pain using a visual analogue scale (VAS), operation time, calf girth, and thigh girth. A random-effects meta-analysis was performed to account for heterogeneity, with weighted mean difference (WMD) and 95% confidence intervals (CI) used as pooled estimates for clinical outcomes.

**Results:**

Of the nine potentially related studies, seven eligible studies (sufficient quantitative data) were included in the meta-analysis. Postoperative drain output in the tourniquet group was on average 100 ml higher than in the non-tourniquet group (95% CI: 36 to 168). Pain, measured by the VAS at 24 h postoperatively, was 0.42 points higher in the tourniquet group (95% CI: 0.08 to 0.76), with the increase persisting at 48 h, averaging 0.40 points (95% CI: 0.12 to 0.69). Thigh girth in the tourniquet group was reduced by 1.8 cm (95% CI: -2.7 to -0.94). No significant differences were observed for calf girth and the operation time.

**Conclusion:**

Our meta-analysis indicates that tourniquet use during arthroscopic ACL reconstruction is associated with higher pain levels, increased postoperative drain output, and reduced thigh girth. However, performing the surgery without a tourniquet does not significantly extend the operation time.

**Trial registration:**

The protocol was registered in the International Prospective Register of Systematic Reviews, PROSPERO (CRD42023417604).

## Background

Anterior Cruciate Ligament (ACL) rupture is common among physically active young individuals. If untreated, it can lead to joint instability and cartilage damage [20]. ACL reconstruction is the preferred treatment for knees with ACL deficiency due to its promising clinical outcomes and enabling a return to sports activities [4, 28]. Initial 1904, tourniquet use has since become routine in orthopedic surgeries [34]. Most orthopedic surgeons use tourniquets to limit blood flow to the surgical site, creating a clear, bloodless field, particularly useful in lower extremity procedures. This approach has been reported to enhance visibility, alleviate technical challenges, improve procedural accuracy, and reduce operation time [15, 22, 34, 36, 37]. However, the application of tourniquets carries inherent risks, including thigh pain, increased analgesic requirements, increased post-operative drain output, and potential for deep-vein thrombosis (DVT) [18, 34]. Additional disadvantages with tourniquet implementation also includes an elevated risk of nerve palsy [21, 29], vascular injury [30], muscle damage [19, 32], delayed rehabilitation [32], as well as post-operative swelling and stiffness [16, 18].

The existing literature provides mixed findings regarding tourniquet use in ACL reconstruction. Some studies have linked tourniquet application with increased postoperative pain, numbness, knee hemarthrosis, and temporary muscle weaknessly [27], tourniquet-assisted arthroscopic knee surgeries have shown benefits such as shorter operation times, improved visualization, and reduced intraoperative blood loss [12, 24, 36]. Systematic reviews exploring the effects of tourniquet use during ACL reconstruction have been published [18, 33, 34]. The reviews conducted by Wu et al. and Kuo et al. concluded that tourniquet usage in ACL reconstruction may not significantly reduce operation time in ACL surgeries [18, 34]. However, owing to the heterogeneity among the included studies and the limited number of high-quality randomized clinical trials, the impact of tourniquet application on both intraoperative and post-operative outcomes remains inconclusive. Furthermore, the clinical significance of the observed increase in drain output, pain scores, and complication rates remains ambiguous. Currently, whether to use a tourniquet in ACL reconstruction is often left to the surgeon’s discretion.

To provide a more comprehensive conclusion regarding the advantages and disadvantages of tourniquet application in arthroscopic ACL reconstruction, we conducted an updated systematic review and meta-analysis. This study aims to evaluate the effects of tourniquet use on post-operative outcomes, including drain output, pain score, thigh, and calf girth, as well as the operation duration.

## Materials and methods

This systematic review and meta-analysis were registered in the International Prospective Register of Systematic Reviews (PROSPERO) (CRD42023417604, https://www.crd.york.ac.uk/PROSPERO/).

### Study eligibility (inclusion and exclusion criteria)

**Fig. 1:**
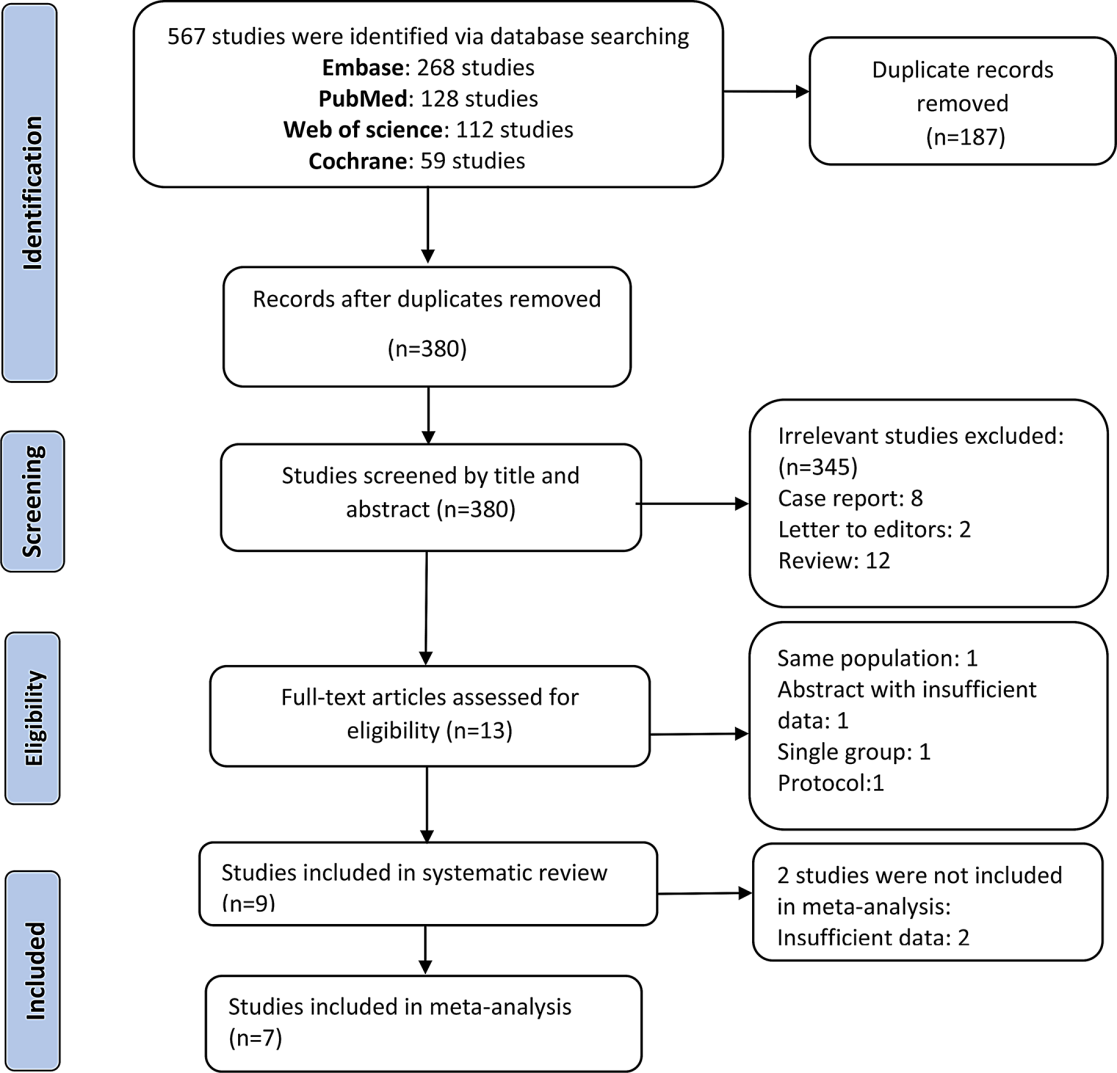
Flowchart of study selection

Studies were considered acceptable for inclusion in the system review if they met the following PICOD criteria: Population (P): arthroscopic ACL reconstruction; Intervention (I): tourniquet application; Comparison (C): no tourniquet application; Outcome (O): surgical outcomes and complications’ rate; Design (D): randomized clinical trial (RCT) or non-randomized studies of intervention (NRSIs). The following criteria were used for the exclusion of trials: lack of reporting on outcomes of interest, non-English language abstracts, and unavailability of data (Fig. 1).

### Literature search

The research protocol for this review was determined by all authors before the literature searches were begun. We searched comprehensively for all controlled clinical trials, either RCT or NRSIs, that compared surgical outcomes or complication rates following tourniquet use against non-tourniquet use during ACL reconstruction surgery. We searched the Cochrane Central Register of Controlled Trials, MEDLINE, Web of Science, and EMBASE for relevant controlled clinical trial from inception to March 3, 2023. The search strategy was conducted by the following mesh and text keywords: (“tourniquet” OR “tourniquets”) AND (“reconstruction” OR “surgery” OR “repair” OR “operations” OR “rehabilitation”) AND (“anterior cruciate ligament” OR “ACL”). The reference lists of included studies were also checked to identify relevant controlled clinical trial (Fig. 1).

### Study selection and data abstraction

Two reviewers (MS and MD) independently extracted the following data: first author, year of publication, sample size, patient’s characteristics (age and gender), use of tourniquet protocol (pressure, time), outcomes (operation time, blood loss through the intra-articular drain, total drain output, thigh girth, calf girth, pain score, quadriceps and hamstring strength) and main findings (effect size and pertinent 95% confidence interval) of each study. A third author (MHE) arbitrated when the two authors disagreed.

### Risk of bias assessment

**Fig. 2:**
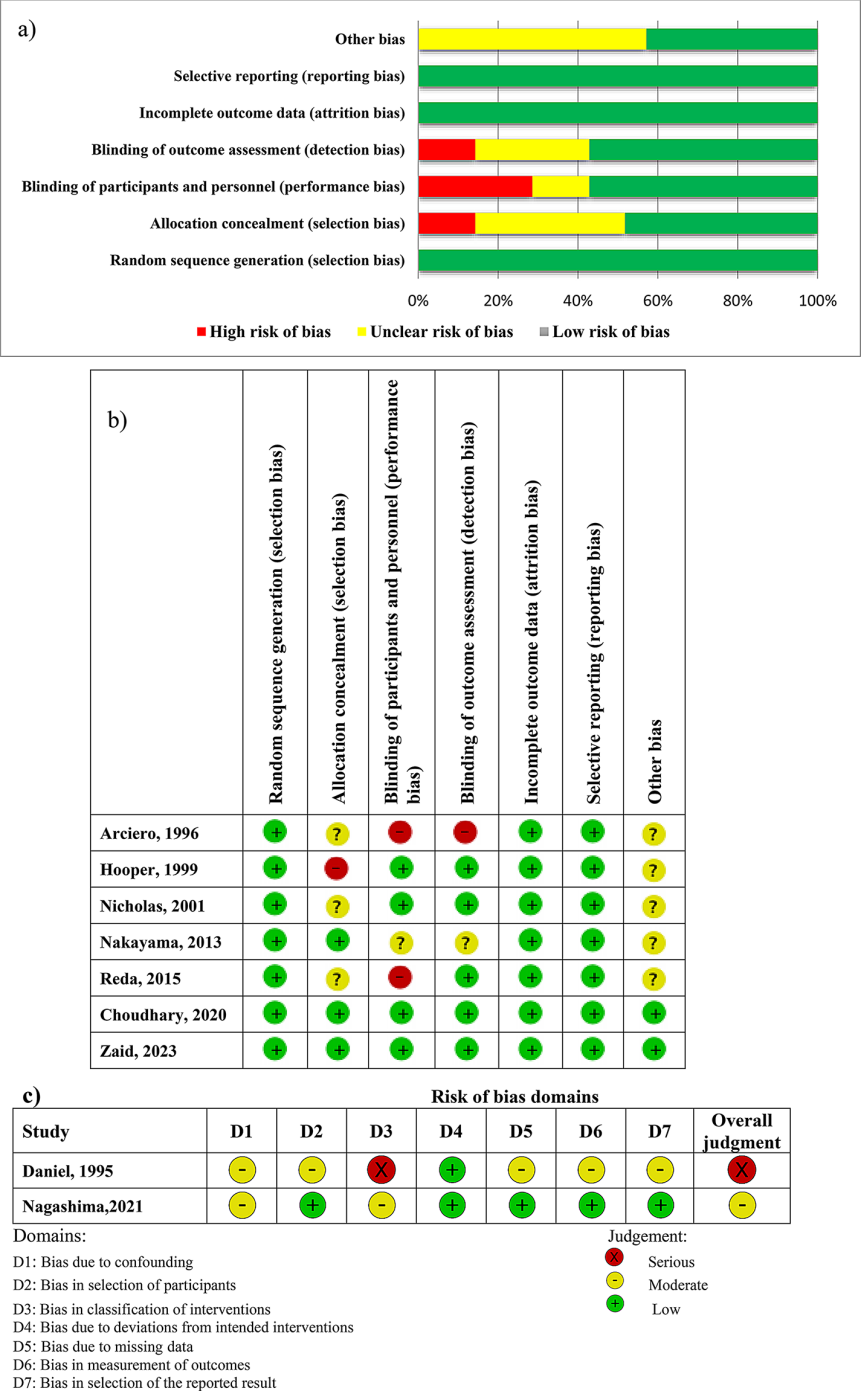
(**a**) Risk of bias graph. (**b**) Risk of bias summary. (**c**) Risk of bias presented using Cochrane ROBINS-I tool for the non-randomized

Assessment of methodological quality of the included clinical trials (RCTs) was conducted by two reviewers using the Cochrane Collaborations risk of Bias tool (ROB) for assessing the risk of bias in randomized trials [2, 6, 13, 24, 25, 27, 36] and Cochrane ROBINS-I tool [7, 23] for Risk of Bias in the NRSIs, respectively. The ROB tool is used to evaluate seven domains of bias: random sequence generation (selection bias), allocation concealment (selection bias), blinding of participants and personnel (performance bias), blinding of outcome assessment (detection bias), incomplete outcome data (attrition bias), selective reporting (reporting bias), and other sources of bias (other bias). A risk of bias judgment was made for the domain as either “low risk of bias”, “unclear risk of bias”, or “high risk of bias”. Finally, an overall judgment of the risk of bias was made for each study according to the Cochrane recommendation (Fig. 2).

Following seven domains of risk were critically evaluated based on ROBINS-I tool: 2 domains before the intervention, 1 at the intervention, and 4 domains after the intervention. This tool considers biases that may arise from confounding, selection of study participants, classification of interventions, deviations from the intended interventions, missing data, outcome measurements, and bias in the selection of the reported results. Each domain was judged as a low, moderate, serious, or critical risk of bias. Two reviewers (MS and MD) assessed the risk of bias in studies and the third reviewer (MS) verified it. Any disagreements in methodological quality assessment were resolved by discussion between reviewers and consultation with a third reviewer (MHE).

### Data analysis

For each controlled clinical trial, the mean, standard deviation, and sample size in both groups (tourniquets versus non-tourniquet) for pre-specified continuous clinical outcomes were extracted and prepared for analysis. Forest plots were depicted to assess for heterogeneity and calculate the pooled weighted mean difference with corresponding 95% confidence intervals (WMD with 95% CI) for visual inspection across studies. Due to clinical and methodological heterogeneity, a random-effects meta-analysis was conducted to account for the heterogeneity of the study populations. Pooled estimates with their corresponding 95% CIs were calculated using inverse-variance weights methods [8]. I^2^ statistics was used to assess the heterogeneity across studies [10] (I^2^ = 0% indicates no observed heterogeneity and I^2^ ≥ 50% indicates substantial heterogeneity). Cochran’s Q statistic was also used to analyze the statistical significance of heterogeneity [11]. Sensitivity analysis was performed to determine which study (if any) had the largest impact on the heterogeneity and to assess the robustness of pooled estimates. Visual inspection of funnel plots was done to assess publication bias [9]; WMD was plotted against the inverse of the square of the standard error. All statistical tests were two-tailed, and the significance level was set at less than 0.05 for all, except for the heterogeneity test. Statistical analyses were performed using Stata version 17.0 (Stata Corp., College Station, TX, USA).

## Results

### Literature search

A total of 567 potentially relevant citations were extracted from the four electronic databases. After excluding 187 duplicates and 345 irrelevant citations based on titles and abstracts, 13 full-text articles were selected for further review. Finally, nine studies published between 1995 and 2023 met the inclusion criteria and were included in the systematic review. The meta-analysis was conducted on seven studies with sufficient quantitative data. The detailed study selection process is illustrated in Fig. 1.

### Study selection and data abstraction

Nine controlled clinical trials with a total of 680 participants fulfilled the inclusion criteria, comprising 342 patients in the tourniquet group and 338 patients in the non-tourniquet group. Tourniquet pressures ranged from 250 to 350 mm Hg, with mean tourniquet times spanning from 10 to 105 min. The mean age of patients ranged from 11 to 45 years. The maximum follow-up period was six months. The study characteristics are detailed in Table 1.

**Table 1:** Main characteristics of the included studies

Author, Year (Ref)	Country	Study design	Sample size (T/NT)	Age (year)		Gender (F/M)		Tourniquet parameters		Study population	Follow up (Day)	Outcome measures
				T	NT	T	NT	Pressure (mmHg)	Time (min)			
Arciero, 1996 [2]	USA	RCT	40 (20/20)	26 (18–41)	22 (18–34)	7/13	3/17	269	87 (64–105)	ACL-deficient knee without Grade III capsular ligamentous injury	28,168,336	Operative time; serum CPK values; thigh and calf girth; isokinetic testing; functional testing
Choudhary, 2020 [6]	India	RCT	45 (23/22)	29.30 ± 11.06	29.64 ± 10.95	2/21	5/17	NA	76.48 ± 8.71	Complete anterior cruciate ligament rupture with instability of the knee	0,1,2,3,4	Thigh girth, CPK values, pain score
Daniel, 1995 [7]	USA	NRSI	94 (48/46)	26	26	12/36	14/32	280	94	ACL-deficient knee indicated for reconstruction	0,42,84,168,364	Quadriceps and hamstring index, Strength testing, hop index, and thigh girth
Nagashima, 2021 [23]	Japan	NRSI	115 (55/60)	30.5 ± 15.1	27.1 ± 12.1	28/27	30/30	250–280	68.5 ± 14.7	ACL-deficient knee indicated for reconstruction	7	Operative time, Blood loss, Deep venous thrombosis
Nakayama, 2013 [24]	Japan	RCT	51 (28/23)	24.8 (14–45)	26.7 (15–45)	17/11	7/16	300	11.8 ± 2.1	anatomic double-bundle ACL reconstructions with hamstring tendon autograft	3,5, 7, 14, 21	Operative time; pain score; numbness score; thigh girth; total blood loss; blood loss through the drain; function testing
Nicholas, 2001 [25]	USA	RCT	48(25/23)	33 ± 7	32 ± 9	12/13	7/16	300	85 ± 7	ACL tear were scheduled for reconstruction using a central one third patella-tendon autograft	0,21,168	Thigh girth; calf girth; dorsiflexion strength; plantarflexion strength
Reda, 2015 [27]	Egypt	RCT	58(29/29)	25.5 ± 4	25 ± 4.6	4/25	5/24	350	64 (± 8.7)	ACL tears with an anatomical single-bundle ACL reconstruction	7,14,28,56,84,112,140,168	Post-operative pain, need for analgesics, the volume of blood obtained in the drain, girth diameter changes in the thigh and calf, muscle strength and amount of haemarthrosis
Zaid, 2023 [36]	China	RCT	200(100/100)	26.8 ± 6.7	25.2 ± 6.8	31/69	29/71	260.7 ± 28.3	NR	ACL reconstruction using a 4-strand hamstring autograft either without augmentation or with Ligament Advanced Reinforcement System (LARS) augmentation	84,168,336	VAS Scores, Opioid consumption, Thigh circumference, Knee circumference, Calf circumference, Serum CPK levels, Hb concentration, Operation time
Hooper, 1999 [13]	Canada	RCT	29(14/15)	35.3 ± 7.8	35.7 ± 6.6	9/5	5/10	300	NR	anterior cruciate ligament reconstruction using semitendinosus tendon graft	NR	Post-operative morphine consumption, post-operation pain score, arthroscopic visibility and satisfaction with pain management

T: Tourniquet; NT: No Tourniquet; RCT: Randomized controlled trial; NRSI: Non-randomized study of intervention; NR: Not reported; NA: Not applicable

Six studies [2, 13, 23, 24, 27, 36] involving a total of 493 patients reported the operation time, while four studies [23, 24, 27, 36] with 424 patients reported post-operative drain output within the first 24 h. Three studies [2, 27, 36] including 298 participants, evaluated the post-operative thigh and calf girth (Table 2). Five studies [6, 13, 24, 27, 36] with a total of 383 patients, assessed patient-reported post-operative pain using VAS scores (Table 3). Choudhary et al. and Reda et al. provided VAS scores at 4, 10, 16, and 22 h post-surgery [6, 27], while Zaid et al. reported VAS scores at 12, 24, and 48 h post-operatively [36].

### Risk of bias assessment

Figure 2 (a, b, c) illustrates the risk of bias across individual studies (seven RCTs and two NRSIs). Of the seven RCTs, risk of bias was low for two studies, and unclear/moderate for five, based on ROB tool. All of the studies expressed the assignment of subjects to the intervention and control groups were random, and provided clear explanations of the random sequence generation method. It seems that all RCTs were deemed to be low risk of attrition and reporting bias. Around 57.15% of the RCTs were considered overall low risk of performance or detection bias, commonly due to blinding of participants and outcome assessors (Fig. 2-a). Daniel et al. [27] was considered to be serious risk of bias, while one NRSI was rated moderate quality (Fig. 2-c).

**Table 2:** Included studies’ outcomes

Author, Year	Post-operative drain output (ml)		Calf girth (cm)		Thigh girth (cm)		Operation time (min)	
	T	NT	T	NT	T	NT	T	NT
Arciero, 1996 [2]	-	-	30.82	31	40.71 ± 3.75	41.74 ± 2.37	128 (85–200)	137 (96–200)
Nagashima, 2021 [23]	201.9 ± 76.9	149.9 ± 60.3	-	-	-	-	80.8 ± 18.7	78.5 ± 15.1
Nakayama, 2013 [24]	133.6 ± 62.4	85.3 ± 47.3	-	-	-	-	115 ± 18	122 ± 17
Reda, 2015 [27]	327.6 ± 57.4	186.7 ± 47.1	30.9 ± 1.8	33.1 ± 3	33.4 ± 1.9	35.6 ± 3	64 ± 8.7	62 ± 9.1
Zaid, 2023 [36]	240.3 ± 44.5	75.6 ± 15.3	38.9 ± 4.8	39.3 ± 5	51.8 ± 6.2	53.6 ± 4.6	58.4 ± 5.7	72.5 ± 8.6
Hooper, 1999 [13]	-	-	-	-	-	-	60.8 ± 9.6	65.3 ± 15.5

T: Tourniquet; NT: Non-Tourniquet

**Table 3:** Included studies’ VAS score results at different time intervals after surgery

Author, Year	VAS score (hours)									
	4–5		10–12		16		22–24		48	
	T	NT	T	NT	T	NT	T	NT	T	NT
Choudhary, 2020 [6]	7.4 ± 1.5	6.1 ± 1.5	8.0 ± 0.9	5.9 ± 1.5	7.4 ± 1.3	5.1 ± 1.1	5.6 ± 0.9	4.6 ± 1.0	2.6 ± 1.0	2.2 ± 1.2
Nakayama, 2013 [24]	-	-	-	-	-	-	2.1 ± 0.7	1.9 ± 0.7	-	-
Reda, 2015 [27]	8.5 ± 1.1	4.9 ± 0.9	5.7 ± 1.3	4.8 ± 1.2	4.8 ± 0.9	4.8 ± 1.1	4.3 ± 0.8	4.2 ± 0.9	-	-
Zaid, 2023 [36]	-	-	5.1 ± 1.2	4.9 ± 1.3	-	-	4.5 ± 1.3	4.0 ± 1.0	3.2 ± 1.0	2.8 ± 1.3
Hooper, 1999 [13]	0.75 ± 0.81	0.45 ± 0.81	-	-	-	-	-	-	-	-

T: Tourniquet; NT: Non-Tourniquet

### Data analysis

The study of Nicolas et al. [25] was not analyzed due to insufficient data, and the study by Daniel et al. [7] was also excluded from the meta-analysis due to both poor quality and insufficient data. Finally, the meta-analysis was conducted on seven out of the total nine articles.

#### Post-operative drain output (ml)

**Fig. 3:**
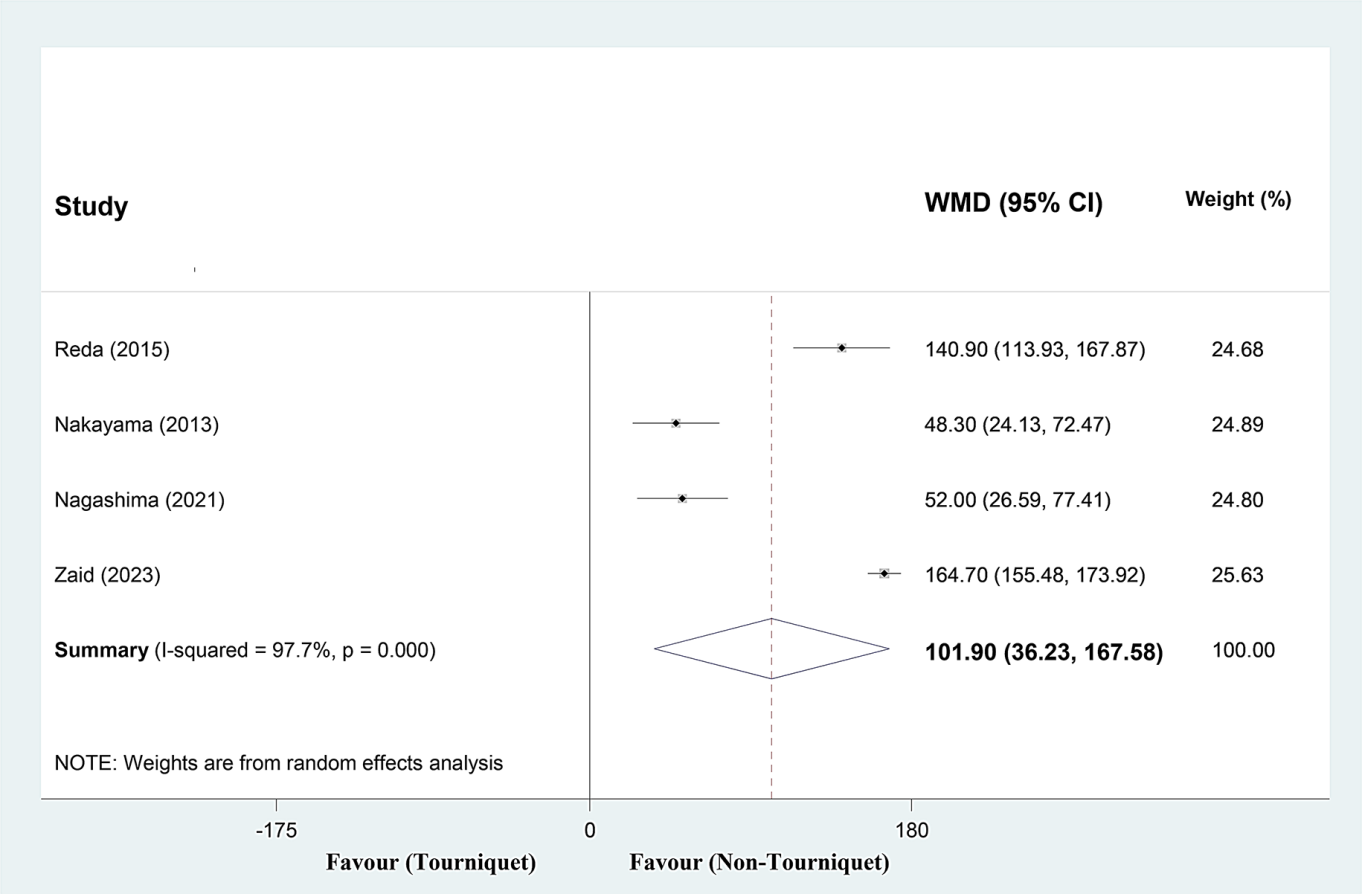
Forest plot of **post-operative drain output (ml)** for tourniquet group versus non-tourniquet group in patients who underwent ACL reconstruction. Diamond represents the summary weighted mean difference (pooled WMD) estimate and its width shows corresponding 95% CI with random effects estimate. I2 test and Cochran’s Q statistic were used to assessing the statistical heterogeneity (*P* < 0.10) across studies

Analyzing four studies, drain output increased a mean of 101.90 ml (range, 36.23 to 167.58) in the tourniquet group (Fig. 3). Sensitivity analysis by successively removing a particular study at a time to assess the influence of every single study on post-operative drain output (ml) showed that the meta-analysis findings was relatively robust (range of summary WMDs: 80.17- 119.79). The I square showed high heterogeneity among reported data for drain output (I2: 97.7%, P 0.000).

#### Pain VAS score

**Fig. 4:**
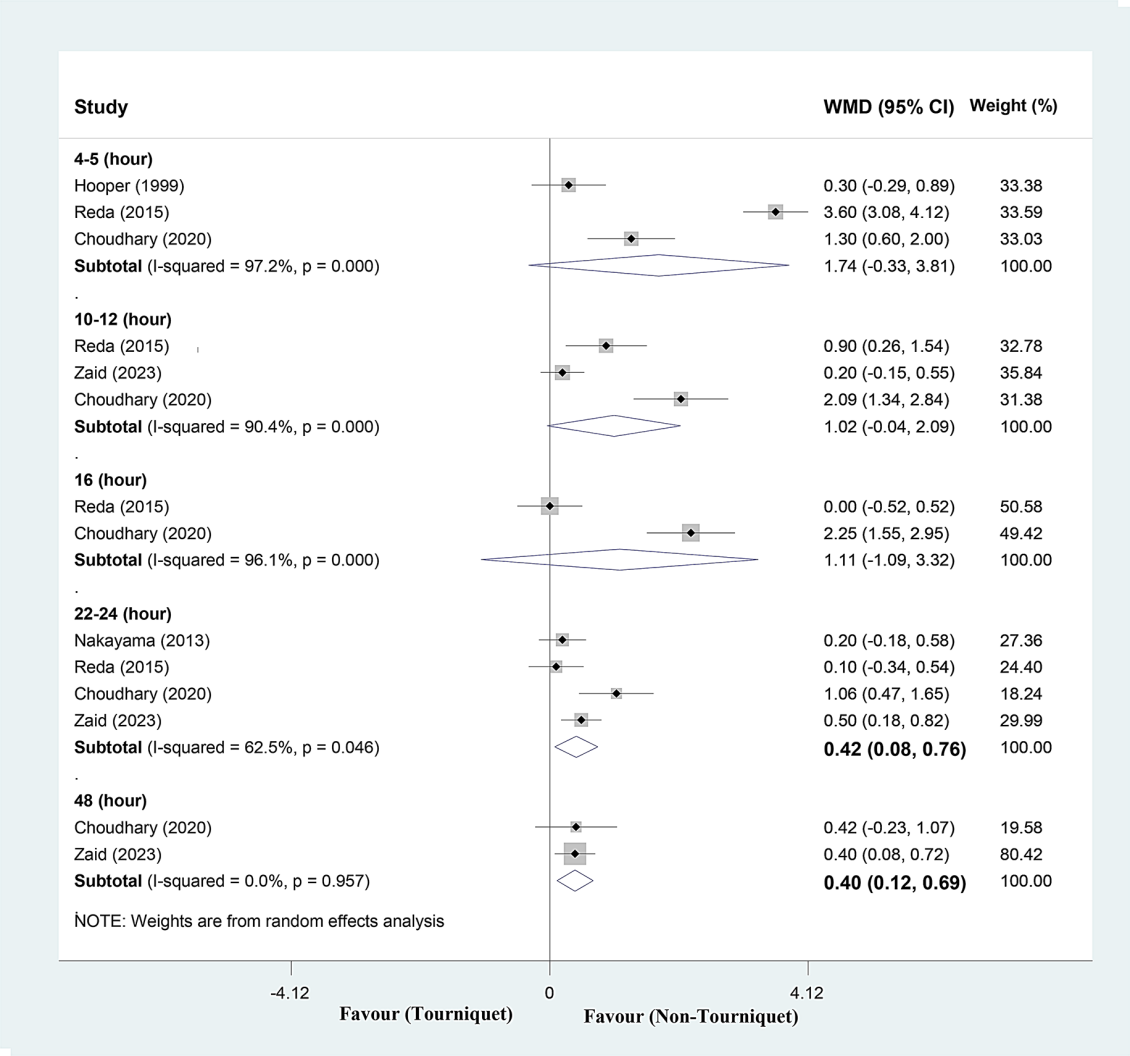
Forest plot of post-operative **pain score (VAS)** for tourniquet group versus non-tourniquet group in different time intervals post-operatively. Diamond represents the summary weighted mean difference (pooled WMD) estimate and its width shows corresponding 95% CI with random effects estimate. I^2^ statistics and Cochran’s Q test were used to assessing the statistical heterogeneity (*P* < 0.10) across studies. Sensitivity analyses were conducted for pain VAS score. Findings showed that weighted mean difference in pain score were consistent at 4–5, 10–12, and 22–24 h post-operative (range of summary WMDs: 0.80, 2.46; 0.50, 1.47; and 0.30, 0.53) respectively, indicating that the meta-analysis models were almost robust

Analyzing three studies, pain VAS score at 4–5 h post-operatively increased a mean of 1.74 scores (range, -0.33 to 3.81), in the tourniquet group (Fig. 4). The I square showed high heterogeneity among reported data for pain VAS score (I^2^: 97%, *P* < 0.001).

Analyzing three studies, pain VAS score at 10–12 h post-operatively increased a mean of 1.02 scores (range, -0.04 to 2.09), in the tourniquet group (Fig. 4). The I square showed high heterogeneity among reported data for pain VAS score (I^2^: 90.4%, *P* < 0.001).

Pooled analysis of two studies, pain VAS score at 16 h post-operatively increased a mean of 1.11 scores (range, -1.09 to 3.32), in the tourniquet group (Fig. 4). The I square showed high heterogeneity among reported data for pain VAS score (I^2^: 96.1%, *P* < 0.001).

Analyzing four studies, pain VAS score at 22–24 h post-operatively increased a mean of 0.42 scores (range, 0.08 to 0.76), in the tourniquet group (Fig. 4). The I square showed moderate heterogeneity among reported data for pain VAS score (I^2^: 62.5%, *P = 0.046* ).

Pooled analysis of two studies, pain VAS score at 48 h post-operatively increased a mean of 0.40 scores (range, 0.12 to 0.69), in the tourniquet group (Fig. 4). The I square not showed heterogeneity among reported data for pain VAS score (I^2^: 0%, *P* = 0.957).

Sensitivity analysis showed the mean change of pain score were consistent at 4–5, 10–12, and 22–24 h post-operative (range of summary WMDs: (0.80, 2.46; 0.50, 1.47; and 0.30, 0.53) respectively, indicating that the meta-analysis models were almost robust.

#### Operation time (minutes)

**Fig. 5:**
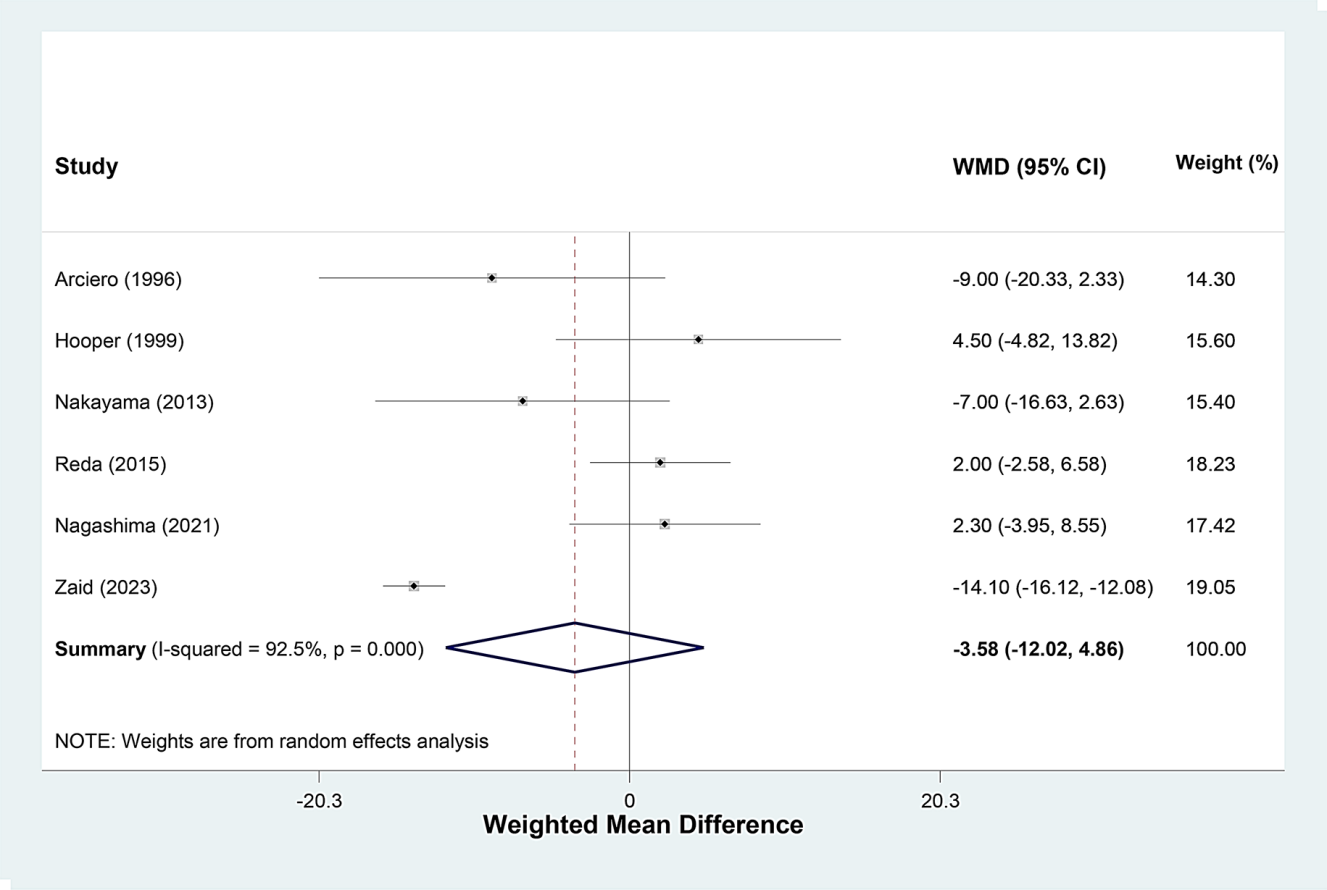
Forest plot of **operation time (min)** for tourniquet group versus non-tourniquet group in patients who underwent anterior cruciate ligament (ACL) reconstruction. Diamond represents the summary weighted mean difference (pooled WMD) estimate and its width shows corresponding 95% CI with random effects estimate. The size of the square and its central point reflects the study specific statistical weight (inverse of variance) and point estimate of the WMD and horizontal line reflects corresponding 95% CI of the study. I^2^ test and Cochran’s Q statistic were used to assessing the statistical heterogeneity (*P* < 0.10) across studies

Analyzing six studies, operation time decreased a mean of 3.58 min (range, -12.02 to 4.86), in the tourniquet group (Fig. 5). Sensitivity analysis by successively removing a particular study at a time to assess the influence of every single study on pooled mean of operative time (minute) showed that the meta-analysis findings was relatively robust (range of summary WMDs: -0.11, -4.80). The I square showed high heterogeneity among reported data for operation time (I2: 92%, *P* < 0.001).

#### Thigh and calf girth (cm)

**Fig. 6:**
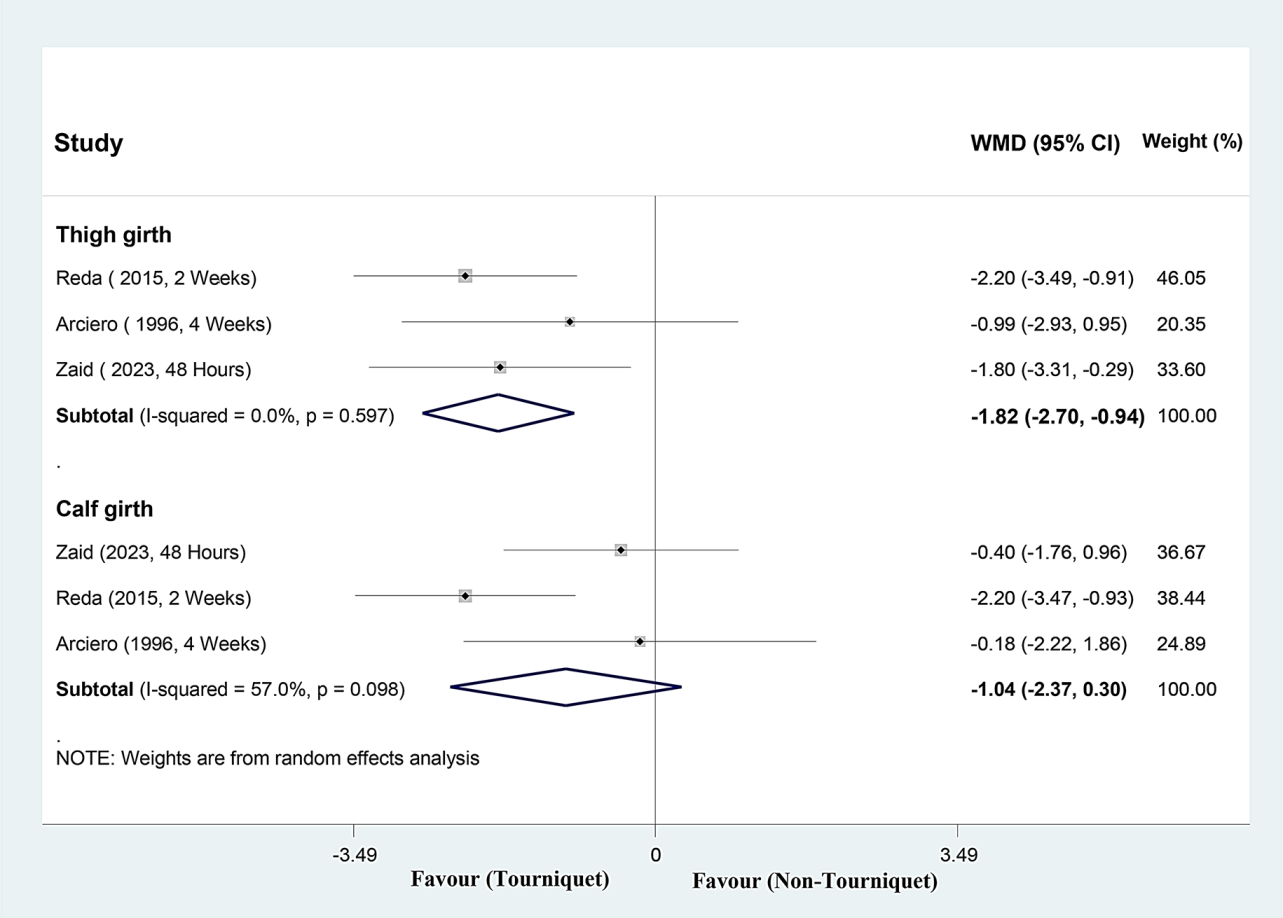
Forest plot of post-operative **thigh and calf girth (cm)** for tourniquet versus non-tourniquet group in patients who underwent ACL reconstruction. Diamond represents the summary weighted mean difference (pooled WMD) estimate and its width shows corresponding 95% CI with random effects estimate. I2 test and Cochran’s Q statistic were used to assessing the statistical heterogeneity (*P* < 0.10) across studies. Weighted mean difference in thigh girth and calf girth were consistent in sensitivity analyses (range of summary WMDs: -2.03, -1.49 and − 1.35, -0.33), which indicated robustness of our findings

Analyzing three studies, thigh girth decreased a mean of 1.82 cm (range, -2.70 to -0.94), in the tourniquet group (Fig. 6). The I square not showed heterogeneity among reported data for thigh girth (I2: 0%, *P* = 0.597).

Analyzing three studies, calf girth decreased a mean of 1.04 cm (range, -2.37 to 0.30), in the tourniquet group (Fig. 6). The I square moderate heterogeneity among reported data for calf girth (I2: 57.0%, *P* = 0.098).

Sensitivity analysis showed the mean change of thigh and calf girth were consistent (range of summary WMDs: (: -2.03, -1.49 and − 1.35, -0.33), which indicated robustness of our findings.

## Discussion

This systematic review and meta-analysis aimed to examine tourniquet use during arthroscopic ACL reconstruction to update clinical evidence and guide surgical decision-making. The primary findings of this study indicate that the use of a tourniquet associates with elevated post-operative drain output and pain scores at both the 24-hour and 48-hour following the operation. Furthermore, a reduction in thigh girth, which is a possible indicator of quadriceps muscle atrophy, was found. On the other hand, no significant difference was identified in terms of operation time and post-operative calf girth.

The existing literature presents conflicting evidence regarding the association of tourniquet use and post-operative pain intensity [18, 36]. Notably, some studies have reported that tourniquet usage exacerbates post-operative pain [5, 27], while others have yielded inconsistent results [13, 18]. Reda et al. conducted a study that demonstrated an increase in post-operative pain and hemarthrosis following ACL reconstruction with tourniquet application [27], which contrasts with the findings of Zaid et al., who observed no significant difference in post-operative pain between the groups [36]. We believe that the discrepancy in results may be attributed to factors such as tourniquet cuff pressure, duration of tourniquet application exceeding 30 min, and tourniquet width [6, 36]. In the present study, a statistically significant difference in post-operative pain was observed between the study groups at the 22-hour time interval following the operation. However, this difference (0.42 scores (range, 0.08 to 0.76)) is not clinically significant, considering the literature typically ranges from 1.0 to 4.0. Recent studies suggest that the MCID for the VAS is about 1.65 to 2.0 on a scale of 0 to 10 [35]. However, at earlier time intervals, where the data also displayed considerable heterogeneity, no statistically significant difference was identified. Our hypothesis is that the subjective nature of pain intensity, coupled with the heightened distress experienced during the early post-operative period, likely contributed to the wide variability observed in the reported VAS pain scores. Consequently, the reliability of the results before 22-hour post-operatively may be limited.

Hyperemia and shunt phenomena following tourniquet release can contribute to inadequate hemostasis and increased drainage output, potentially causing discomfort during post-operative rehabilitation exercises [1, 18, 36]. To address this concern, the use of a pressure dressing prior to tourniquet release has been observed to effectively stop minor bleeding from vessels [14]. Despite the established association between a shorter duration of surgery and a reduced risk of adverse events such as surgical site infections, readmissions, sepsis, deep vein thrombosis (DVT), and hospital stays [31, 36], our findings are in line with previous meta-analyses [18, 33, 37], that indicate no statistically significant difference in operation time between the tourniquet and non-tourniquet groups. Muscle weakness and atrophy are another concerns related to tourniquet use [18]. Several studies have demonstrated that tourniquet application is associated with prolonged muscle weakness and delayed functional recovery [26, 27]. Consistent with these findings, our research has confirmed a significant decrease in thigh girth, a possible indicator of quadriceps muscle atrophy, associated with tourniquet utilization. Similarly, several other studies have reported significant differences in thigh girth measurements between the tourniquet and non-tourniquet groups [2, 6, 7, 25, 27]. However, contrasting views have been presented by Zaid et al. and Nakayama et al., suggesting that tourniquet application has no noticeable effect on thigh girth [24, 36]. A systematic review on this topic reported that intraoperative tourniquet use resulted in decreased thigh circumference and detrimental EMG changes in quadriceps function in 3 of the 5 included studies [3]. Even when compared between low and high pressure tourniquet use, the high pressure group had negative EMG changes post-operatively [17].

We observed significant heterogeneity among the studies, indicated by high I-square values. Due to the high heterogeneity, subgroup meta-analyses were conducted. Only in VAS scores, follow-up time explains the observed heterogeneity. Our hypothesis suggests that multiple factors may contribute to the substantial heterogeneity and high I² values in the reviewed studies. When interpreting study results, it is important to consider that various factors may influence outcomes.

One primary source of heterogeneity identified in our meta-analysis is clinical variability, which cannot be fully controlled with statistical methods. For instance, different anesthesia techniques can impact operative times and contribute to the high heterogeneity observed. We also noted considerable variation in surgical techniques, particularly in methods for ACL reconstruction, such as single-bundle versus double-bundle techniques, which can affect surgical complexity and duration. Tourniquet pressures also varied widely across studies (from 250 to 350 mm Hg), as did application duration (10 to 105 min), potentially impacting intraoperative blood loss and drain output.

Additionally, the quality of studies included in the meta-analysis varied significantly. Differences in methodological aspects, such as randomization processes, blinding, and sample sizes, can introduce further variability in results. Some studies were rated as having serious risks of bias, which could affect outcome reporting and interpretation. Although a random-effects model was used to account for this heterogeneity, clinical variability remains an inherent challenge in surgical studies. Future research should aim for standardized methodologies, consistent measurement of surgical outcomes, and diverse study populations to enhance the generalizability of results across clinical settings.

This study represents the most recent systematic review and meta-analysis of controlled CTs aimed at examining the effects of tourniquet use in arthroscopic ACL reconstruction. However, caution is needed when applying the results of these studies to clinical practice, given the limited number of RCTs, the inclusion of some outdated studies, and the resulting heterogeneity in clinical settings and outcome assessments across studies. To address this limitation, larger-scale, multi-center RCTs may be necessary to provide further clarification on this matter. Furthermore, as the use of postoperative drains in ACL reconstructions has significantly decreased and is no longer common practice, additional clinical trials measuring intraoperative and postoperative blood loss using current practical tools are needed. Compared to drainage volume, hemoglobin levels or calculated total blood loss are preferable for assessing intraoperative blood los.

## Conclusion

Our study indicates that the use of a tourniquet during arthroscopic ACL reconstruction is associated with increased post-operative drain output and greater pain intensity after 22 h, as well as a reduction in thigh girth, compared to surgeries performed without a tourniquet. These findings suggest that avoiding tourniquet use could improve early post-operative outcomes by reducing patient discomfort and limiting post-operative swelling. Furthermore, the lack of significant impact on operation time when omitting the tourniquet suggests that this approach does not compromise surgical efficiency. For clinicians, these findings support considering non-tourniquet procedures in ACL reconstruction. Future research should focus on larger, multi-center trials to further validate these findings and explore long-term functional outcomes, such as thigh muscle recovery and overall rehabilitation timelines, to provide more robust evidence for surgical guidelines.

## Data Availability

The datasets used and/or analysed during the current study are available from the corresponding author on reasonable request.
